# What you see is not what you get: implications of the brevity of antibody responses to malaria antigens and transmission heterogeneity in longitudinal studies of malaria immunity

**DOI:** 10.1186/1475-2875-8-242

**Published:** 2009-10-28

**Authors:** Samson M Kinyanjui, Philip Bejon, Faith H Osier, Peter C Bull, Kevin Marsh

**Affiliations:** 1 Kenyan Medical Research Institute (KEMRI), Centre for Geographic Medicine Research (Coast), PO Box 230, Kilifi 80108, Kenya; 2 Nuffield Department of Medicine, Centre for Clinical Vaccinology and Tropical Medicine, University of Oxford, Churchill Hospital, Oxford, UK

## Abstract

**Background:**

A major handicap in developing a malaria vaccine is the difficulty in pinpointing the immune responses that protect against malaria. The protective efficacy of natural or vaccine-induced immune responses against malaria is normally assessed by relating the level of the responses in an individual at the beginning of a follow-up period and the individual's experience of malaria infection or disease during the follow-up. This approach has identified a number of important responses against malaria, but their protective efficacies vary considerably between studies.

**Hypothesis:**

It is likely that apart from differences in study methodologies, differences in exposure among study subjects within each study and brevity of antibody responses to malaria antigen are important sources of the variation in protective efficacy of anti-malaria immune responses mentioned above. Since malaria immunity is not complete, anyone in an area of stable malaria transmission who does not become asymptomatically or symptomatically infected during follow-up subsequent to treatment is most likely unexposed rather than immune.

**Testing the hypothesis:**

It is proposed that individuals involved in a longitudinal study of malaria immunity should be treated for malaria prior to the start of the study and only those who present with at least an asymptomatic infection during the follow-up should be included in the analysis. In addition, it is proposed that more closely repeated serological survey should be carried out during follow-up in order to get a better picture of an individual's serological status.

**Implications of the hypothesis:**

Failure to distinguish between individuals who do not get a clinical episode during follow-up because they were unexposed and those who are genuinely immune undermines our ability to assign a protective role to immune responses against malaria. The brevity of antibodies responses makes it difficult to assign the true serological status of an individual at any given time, i.e. those positive at a survey may be negative by the time they encounter the next infection.

## Background

A major handicap in developing a malaria vaccine is the difficulty in pinpointing the responses involved in immunity to malaria and their target antigens [1-3]. The classic approach for assessing the efficacy of natural or vaccine-induced immune responses in protection against malaria is to relate an individual's level of these responses at the beginning of a follow-up period and experience of malaria infection or disease during the follow-up. Using this approach responses against a number of malaria antigens have been shown to be associated with protection against malaria but the strength of these association vary considerably between studies [4-9]. These variations may, in part, be due to differences in methodology, polymorphism of target antigens or epitopes and other factors, such as variation in transmission and exposure [10].

In addition, some of the assumptions inherent in this approach have implications for the interpretation of results of such longitudinal studies. The first assumption is that immune responses observed in an individual at the time of a baseline survey persist throughout the follow-up period (i.e. they provide a stable measure of immune competence) and the second is that we can accurately distinguish "immune" from "susceptible" individuals based on their disease experience during a given period. The discussion below illustrates why these assumptions may be flawed.

## Brevity of antibody responses to malaria antigens

**Figure 1:**
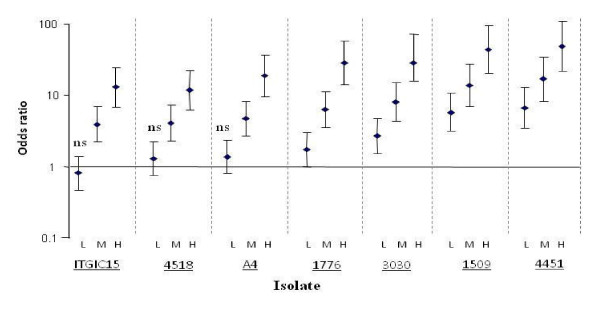
**Age-corrected odds ratios of children having low (L), medium (M) or high (H) levels of antibodies to VSA of various malaria parasite isolates if the children were parasite positive at the time their serum was assayed compared to those who were not**. The odd ratios of having medium or high levels were significantly greater than 1 in all case (P > 0.01). Error bars indicate 95% confidence interval, ns -not significant.

Among people living in endemic areas, levels of antibodies to many malaria antigens may vary with the seasonality of malaria transmission, often being higher during periods of high malaria transmission than at the end of a low transmission season [11-15]. Second, levels of antibodies to malaria antigens often tend to be higher in individuals who also have malaria parasites at the time when their antibodies are measured than in those without parasites [16-18] (Figure 1). These phenomena are typically seen in young children, probably because adults typically have much higher antibody levels that take longer to decay appreciably even in the absence of an infection [12,19,20]. These observations and those from other longitudinal studies [12,21,22], where malaria antibodies fell from relatively high levels to low levels within a few weeks of treatment of a clinical episode, suggest that antibody responses to many malaria antigens are short-lived.

**Figure 2:**
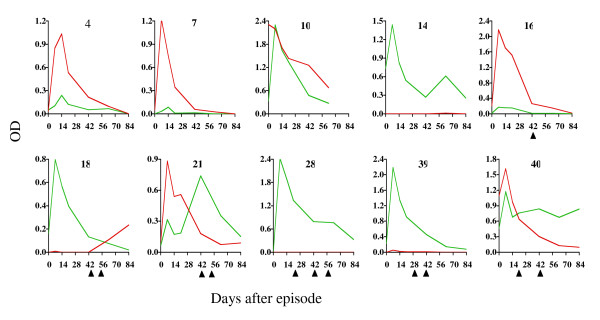
**Antibody responses profiles to two types of MSP2 (red - A type and green - B type) among ten children illustrating the rapid rate of antibody decay following an acute episode of malaria**. The triangles under the graphs indicate time points during follow-up at which parasites were detected.

Recent studies at Kilifi, Kenya confirmed the brevity of responses to several malaria merozoite antigens (MSP1, MSP2, EBA-175 and AMA-1) by closely monitoring levels of IgG antibodies to the antigens over a period of 12 weeks among 42 Kenyan children recovering from an acute episode of malaria [23]. The majority of responses peaked one week after the episode and then decayed rapidly to very low levels in six to eight weeks (Figure 2). Although rapid re-infection limited the ability to make reliable estimates of the half-life of many of the responses, where estimation was possible, IgG1 and IgG3 responses had a mean half-life of about ten and six days, respectively, periods that are shorter than those normally described for the catabolic elimination of these subclasses of antibody. Furthermore re-infection failed to significantly boost the responses [24].

A full discussion on the possible mechanisms underlying the brevity of anti-malaria antibody is beyond the scope of this paper. It has been suggested that the brevity is partially attributable to the predilection of the responses towards short-lived IgG3 [25-28]. However, the study cited above showed that IgG1 to malaria antigens responses are also short-lived [23]. Other possible reasons cited include poor development of malaria antigen-specific memory and long-lived plasma cells [29-31]. However, it suffices to say that the mechanisms are still poorly understood, and none of these mechanisms would explain a rate of decay more rapid than the catabolic half-life of the antibody.

## Heterogeneity of exposure to malaria

Disease incidence is not homogenously distributed within an endemic population. Mathematical modelling of transmission data from different geographic sites by Woolhouse *et al *suggested that about 20% of endemic populations bears 80% of the burden of leishmaniasis, schistosomiasis, sexually transmitted diseases and malaria disease [32]. Subsequent modelling of a set of data that combined malaria measurements from more than 90 communities around Africa showed that indeed, the relationship between community incidence of disease and rates of infectious bites was best explained by a model that incorporated a similar level of heterogeneity in infection [33,34]. The inferences from these models are supported by field data from several places. In a one year study monitoring malaria cases among six villages in northern Ethiopia, 50% of malaria cases occurred in 18% of the households under study while in Belize among 200 households monitored over a period of seven years, 8% the households accounted for 50% of cases detected [35]. GIS based-mapping of distribution of malaria cases in Kampala, Uganda found that local high transmission clusters with a total of 43 children living in them accounted for 22% of cases observed over a two year follow up period despite accounting for only 6.7% of the person-time of observations for the cohort [36]. Similar clustering of malaria has been observed among children resident within a single area of Kilifi, Kenya. Some of the children had a discrepancy of two or more between the numbers of observed and expected episodes of malaria during follow up. 18% of children fell into this group, more than expected by chance alone. At the other extreme, there was a group of children that had neither clinical malaria nor parasites at six cross-sectional surveys during a four-year period [37]. Further observations from Daraweesh, an area of low transmission in Sudan, where over 32 percent of individuals did not suffer a malaria episode over a period of 11 years while others suffered up to eight episodes underlines the variations in individual susceptibility to malaria episodes [38].

A major reason for this heterogeneity appears to be variations in local transmission intensity. The commonly used low resolution malaria endemicity maps often show large areas as having homogenous intensity of transmission while in reality relatively high level of heterogeneity in transmission occur even within very short distances [39,40]. Through fine resolution mapping using GPS, proximity to mosquito breeding sites, such as rivers, dams, vegetation clusters and even temporary pools in wheel tracks, has been shown to play a major role in determining local heterogeneity in transmission [35,40-43]. Studies in The Gambia [42] and elsewhere [35] indicated that transmission intensity falls dramatically a short distance from breeding sites. Importantly clusters of high transmission are not necessarily spatially fixed and may shift over time depending on variations in environmental factors that affect breeding sites [41,44].

In addition to proximity to breeding site, there may be host-related factors that contribute to heterogeneity in the distribution of disease incidence. The finding that in Kilifi familial relationship (after adjusting for shared household) may explain up to a third of the variability in malaria disease experience [45] suggests that in addition to the known malaria resistance genotypes, such as sickle cell trait, there may be a large number of unidentified genes that contribute to variations in individuals' inherent susceptibility to malaria. Furthermore, modeling of human-mosquito contact rates suggest that the contact is not purely random [46], possibly because of inherent variations in host attractiveness to mosquitoes [47]. This means that even individuals who share the same residence might nonetheless be at different risks of receiving infected bites.

## Implication of antibody brevity and heterogeneity in longitudinal studies of malaria immunity

**Figure 3:**
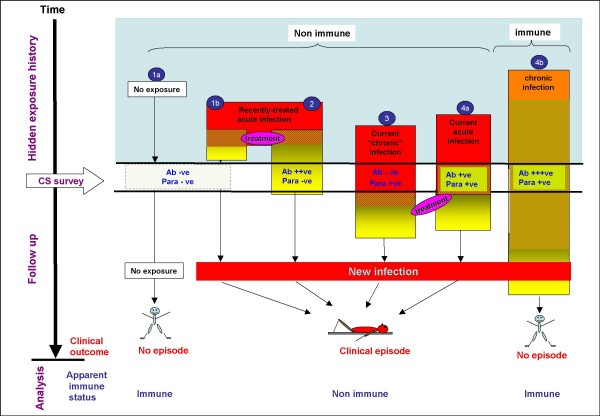
**The influence of exposure history on an individual's apparent serological and parasitological status at a cross-sectional survey (CS) and the individual's clinical history subsequent to the CS**. Ab +ve/-ve = antibody positive or negative, Para +ve/-ve = parasite positive or negative. The red and orange represent infection while yellow represents antibody positivity.

Clearly both brevity of antibody responses and transmission heterogeneity have important implications for longitudinal malaria immunity studies. Figure 3 illustrates how these two factors hinder attempts to define an individual's true malaria immunity status in a longitudinal framework. First, because of these factors, a cross-sectional survey is inadequate as a window into an individual's malaria exposure history. As shown in the figure, the two serological categories of individuals (antibody negative and positive) apparent during a cross-sectional survey are not homogenous groups, yet classical analysis treats them as such because it is not possible to distinguish the various subgroups within them. Within the antibody negative group are individuals that lack antibodies because they are under very low or no exposure (group 1a). Other individuals in the same group are those under exposure but who either did not mount detectable responses to recent exposure (group 3) or if they did, the responses were weak have since decayed (group 1b). On the other hand, the antibody positive group consist of non-immune people with recently treated (group 2) or current acute infection to which they are making responses (group 4a) or immune individuals (group 4b) harbouring chronic infections that may in turn help maintain the high levels of responses. Variations in the proportions of these subgroups in different endemicity settings might in part be the explanation for the differences in the strength of association between antibodies and protection reported by different studies.

Second, even antibody responses that are "protective" might too brief to protect throughout the follow-up period (groups 2 and 4a). In such a case the antibodies would serve only as a makers of exposure. Relative to non-exposed individuals (group 1), the exposed groups, antibody levels notwithstanding, are more likely to eventually present with a clinical episode of malaria leading to the paradoxical conclusion seen in a number of studies (Table 1) that antibodies are associated with increased risk of clinical malaria. Finally, the figure shows that since both non exposure (group 1) and the genuine immunity (group 4b) leads to similar outcome, including the unexposed group in analysis will undermine assignment of a protective role to immune responses. This may help explain why in several recent studies (Table 1), we were able to assign a protective role for antibodies against various malaria antigens only among children who were asymptomatically infected at the time of the serological survey. In other words, having the infection at the survey marked out children that were under some level of exposure and in whom therefore lack of clinical disease during the follow-up most likely reflected immunity.

**Table 1:** The influence of parasitological status on the association between the risk of clinical episode of malaria and antibodies against various malaria antigens

**Study population [ref]**	**Antigen**	**Parasites alone**	**Antibodies alone**	**Parasites + Antibodies**
Chonyi [50]	AMA1 Pro/DI/II/III	**2.65 (P < 0.050)**	1.87 (P = 0.093)	**0.38 (P < 0.050)**
Chonyi [50]	AMA1 DI/II/III		**2.12 (P = 0.049**)	**0.049 (P < 0.050)**
				
Chonyi [7]	MSP2 type A	**2.95 (P < 0.050)**	**8.94 (P = 0.008)**	0.47 (P = 0.414)
Chonyi[7]	MSP2 type B	**3.97 (P < 0.050)**	0.76 (P = 0.599)	0.83 (P = 0.818)
				
chonyi [51]	MSP3		1.14 (P = 0.717)	**0.41 (P = 0.011)**
				
Ngerenya[52]	VSA (isolate A4U)	**3.78 (P = 0.022)**	1.35 (P= 0.567)	1.67 (P = 0.325)
	VSA (isolate A4 40C)	**5.35 (P = 0.005)**	2.88 (P = 0.064)	1.55 (P = 0.402)
	VSA (isolate 3D7)	**7.10 (P = 0.004)**	2.01 (P = 1.700)	1.43 (P= 0.479)
	VSA (isolate P1)	**2.53 (P = 0.035)**	**3.72 (P = 0.050)**	1.38 (P = 0.587)
				
**Antibody responses stratified by titre**				
				
	**Variant surface antigens**			
Ngerenya [18]	Medium tertile	**5.90 (P = 0.018)**	**2.05 (P = 0.06)**	**0.21 (P = 0.200)**
	Highest tertile		**4.34 (P = 0.01)**	**0.09 (P = 0.041)**
				
	**MSP2 type A**			
Chonyi [7]	Lowest quartile	**2.95 (P < 0.050)**		**4.27 (P = 0.050)**
	Highest quartile			0.56 (P = 0.418)
Ngerenya [7]	Lowest quartile	**1.66 (P < 0.050)**		1.11 (P = 0.740)
	Highest quartile			**0.39 (P = 0.044)**
				
	**MSP2 type b**			
Chonyi [7]	Lowest quartile	**3.97 (P < 0.050)**		1.31 (P = 0.530)
	Highest quartile			**0.19 (P = 0.013)**
Ngerenya [7]	Lowest quartile	**1.58 (P < 0.050)**		1.37 (P = 0.547)
	Highest quartile			0.78 (P = 0.560)

Age adjusted risk of suffering a clinical episode of malaria among children in two areas of Kilifi, Kenya (Ngerenya - low transmission and Chonyi- moderate transmission) during follow-up depending on their anti-malarial antibody serological and malaria parasitological status at pre-follow-up cross-section survey. Significant associations (P < 0.05) are highlighted in bold. AMA 1 (Pro/DI, II, III) -Apical Membrane Antigen 1 prodomain and domains 1, 2, and 3. MSP2/3- Merozoite Surface Protein 2/3, VSA - Variant Surface Antigen.

## Dealing with heterogeneity in exposure and brevity of responses in longitudinal studies

The discussion above indicates the need for study designs and analysis approaches that can circumvent the problem of heterogeneous exposure and unstable measures of the responses. One way of correcting for differences in exposure is to use levels of antibodies to total schizonts extract as a proxy of exposure. This approach assumes a linear relationship between exposure and antibody levels. However, this may not be the case partly because of antibody saturation after repeated exposure, seasonal variation, and variations in individual's inherent capacity to respond to malaria antigens. In addition, if measured at the beginning of follow-up, antibodies against schizonts extract reflect past exposure, but not necessarily exposure during the follow-up. Repeated measurement during the follow-up period could help circumvent this problem and give a better picture of the exposure the child faced during this period.

While the incidence of clinical malaria among endemic populations falls with age, the prevalence of parasitization typically rises to a plateau, which is maintained to early adulthood, and even among older adults a substantial proportion is asymptomatically infected at any given time. This suggests that the immunity to malaria acquired with age, while down regulating the severity of infections, does not mediate complete resistance to infection. As such, the ability to harbour an infection asymptomatically rather than having no infection at all might be a more reasonable proxy for immunity in longitudinal studies. The corollary to this being that in endemic areas, individuals who fail to get re-infected within a given period after radical malaria treatment may be more likely to be unexposed rather than immune to re-infection. Therefore carrying out radical treatment prior to follow-up and then doing cross-sectional parasitological surveys within three months to six months of the treatment might be a good way of distinguishing between truly immune individuals and those who are simply unexposed. Those found at the survey to have asymptomatic infection but did not suffer a clinical episode during follow-up can be considered to be immune while those who suffered a febrile episode during the follow-up are considered susceptible. Individuals who have no parasites at the second survey and who did not present with an episode during follow-up should be considered potentially unexposed and left out of analysis. Indeed, in a longitudinal study in Kilifi, which followed this approach the effects of age, anti-VSA antibody responses and transmission intensity were most evident when children who remained uninfected during follow-up (i.e. neither asymptomatic nor febrile infection) in the analysis were excluded in the analysis [48].

An assumption in this approach is that most asymptomatic infections last for several months rather than days. In other words, only in a small proportion of the population will asymptomatic infections during follow-up be so brief as to terminate before the cross sectional survey and therefore cause those individuals to be erroneously considered as unexposed and excluded from analysis along with those who were genuinely unexposed. Observation from malariotherapy work, where non-immune individuals were deliberately infected with malaria parasites, suggest that untreated infections can last for up to nine months with an average of over three months [49].

While very closely space serological surveys would be the ideal way of dealing with the difficulty raised by brevity of potentially "protective" antibody responses, both logistic and ethical constrains makes it difficult carry out too closely spaced surveys. Based on the observed decay profiles of anti-malarial antibodies [23], a serological survey six weeks after the initial bleed (and half way through the follow-up period) would be sufficient for more accurate monitoring of an individuals serological status during follow-up and placing the measurement closer to any episode of clinical malaria they may have.

## Conclusion

Conflicting results concerning the protective efficacy of antibodies against putative vaccine candidate antigens makes it difficult to interpret their relevance to understanding immunity to malaria. In this paper, two factors that might undermine the ability to detect protective antibody responses to malaria antigens in a classical longitudinal studies framework namely the brevity of antibody responses to malaria and heterogeneity in exposure have been discussed. A modification of the framework to include repeated closely spaced surveys to account for antibody decay and radical treatment before the start of follow-up in order to distinguish between genuinely immune individuals and those who are simply unexposed is proposed.

## Competing interests

The authors declare that they have no competing interests.

## Authors' contributions

This paper is a product of discussions between the authors, all of who have been involved in longitudinal studies of malaria immunity at Kilifi, Kenya. SMK prepared the manuscript. All the authors have read and approved the final manuscript.
